# Costs of major depression covered / not covered in British Columbia, Canada

**DOI:** 10.1186/s12913-023-10474-y

**Published:** 2023-12-20

**Authors:** Sonya Cressman, Shahzad Ghanbarian, Louisa Edwards, Sandra Peterson, Mary Bunka, Alison M. Hoens, Linda Riches, Jehannine Austin, Rohit Vijh, Kimberlyn McGrail, Stirling Bryan

**Affiliations:** 1 The Centre for Clinical Epidemiology and Evaluation, Vancouver Coastal Health Research Institute, University of British Columbia, Vancouver, BC Canada; 2 The School of Public and Population Health, University of British Columbia, Vancouver, BC Canada; 3 Faculty of Health Sciences, Simon Fraser University, Burnaby, BC Canada; 4 Centre for Health Services and Policy Research, University of British Columbia, Vancouver, BC Canada; 5 Department of Physical Therapy, University of British Columbia, Vancouver, BC Canada; 6 Patient Partner, University of British Columbia, Vancouver, BC Canada; 7 Department of Medical Genetics, University of British Columbia, Vancouver, BC Canada; 8 Department of Psychiatry, University of British Columbia, Vancouver, BC Canada; 9 Department of Family Practice, University of British Columbia, Vancouver, BC Canada

**Keywords:** Depression, Financial hardship, Financial protection, Inequality, Societal costs, Expenditure proportion, Patient costs

## Abstract

**Background:**

Major depressive disorder (MDD) is one of the world’s leading causes of disability. Our purpose was to characterize the total costs of MDD and evaluate the degree to which the British Columbia provincial health system meets its objective to protect people from the financial impact of illness.

**Methods:**

We performed a population-based cohort study of adults newly diagnosed with MDD between 2015 and 2020 and followed their health system costs over two years. The expenditure proportion of MDD-related, patient paid costs relative to non-subsistence income was estimated, incidences of financial hardship were identified and the slope index of inequality (SII) between the highest and lowest income groups compared across regions.

**Results:**

There were 250,855 individuals diagnosed with MDD in British Columbia over the observation period. Costs to the health system totalled >$1.5 billion (2020 CDN), averaging $138/week for the first 12 weeks following a new diagnosis and $65/week to week 52 and $55/week for weeks 53–104 unless MDD was refractory to treatment ($125/week between week 12–52 and $101/week over weeks 53–104). The proportion of MDD-attributable costs not covered by the health system was 2-15x greater than costs covered by the health system, exceeding $700/week for patients with severe MDD or MDD that was refractory to treatment. Population members in lower-income groups and urban homeowners had disadvantages in the distribution of financial protection received by the health system (SII reached − 8.47 and 15.25, respectively); however, financial hardship and inequities were mitigated province-wide if MDD went into remission (SII − 0.07 to 0.6).

**Conclusions:**

MDD-attributable costs to health systems and patients are highest in the first 12 weeks after a new diagnosis. During this time, lower income groups and homeowners in urban areas run the risk of financial hardship.

**Supplementary Information:**

The online version contains supplementary material available at 10.1186/s12913-023-10474-y.

## Background

Challenges to mental health have a human, social and economic cost that is estimated to exceed $50 billion per year in Canada [1]. Health systems pay for mental health treatments through visits to physicians, emergency departments, hospital admissions and pharmaceuticals; other costs are paid by members of society, external to the health system [2]. It is of particular interest to policy-makers to understand expenditures attributed to one of the most common mental illnesses, Major Depressive Disorder (MDD). Over the next decade, MDD is anticipated to become the world’s leading cause of disability [3]. Since the condition has an adverse impact on productivity, it is forecast that every dollar invested in MDD treatment returns twice as much to the economy [4].

Access to the psychological and pharmacotherapy treatments recommended by the Canadian Network for Mood and Anxiety Treatments (CANMAT) can mitigate the financial impact of MDD [5]. The costs of first-line psychotherapy or pharmacotherapy treatments recommended by CANMAT, however, are not fully covered for everyone in Canada [6, 7]. Most employer-based private insurance plans cover some of these costs, though only two-thirds of Canada’s population have these extended benefit plans [8]. Since MDD also affects a person’s ability to work, people with this condition face additional disadvantages in accessing treatments related both to income and limitations on disability insurance coverage. The implication is that the costs of MDD include direct medical expenditure by public health systems, plus costs covered by private insurance, plus out-of-pocket payments for drugs and other services needed by people with MDD, plus the social and economic impact of people with MDD not being able to engage in their usual activities. Previous studies suggest that economic evaluations of MDD should include productivity changes attributable to the illness, and its treatment [9].

If the proportion of MDD-attributable costs covered by patients becomes too high, patients with the condition are at risk of financial hardship [10]. The World Health Organization (WHO) defines a health system’s ability to provide financial protection as the ratio of an individual’s health-attributed spending in proportion to their non-subsistence income (i.e., net income after accounting for the basic costs of living). If the expenditure proportion for out-of-pocket spending to non-subsistence income exceeds 40%, health systems in middle and high-income countries fail to meet the core objective of providing financial protection [11]. Since not all MDD treatments are covered in Canada, and the condition directly affects the ability of patients to do their paid and unpaid work, we aimed to characterize cost sharing between patients and families managing MDD and British Columbia’s publicly-funded health system.

## Methods

### Overview

The study was part of a multi-component project aimed at evaluating novel treatments for MDD. Our focus was to inform the economic evaluation—in particular, a comprehensive account of the costs attributed to MDD. We used administrative data to calculate costs and median regional individualized income. We convened a Stakeholder Engagement Group (SEG), comprised of patients, decision-makers, pharmacists, clinicians, mental health professionals and caregivers to define MDD-attributable costs paid by patients and guide the development of a literature search to estimate the proportion of MDD-attributable costs not covered by the provincial health system. The expenditure proportion of patient-paid, MDD-attributed costs was determined relative to individualized non-subsistence income, in quintiles. The distribution of financial protection was determined from the slope index of inequality (SII), calculated as the coefficient from linear regression models for the expenditure proportion (outcome) over the lowest to highest income quintiles (ranking fractions) for each region.

### Health system costs covered

#### Data sources

Data were available from the BC Ministry of Health and Statistics Canada and provided by Population Data BC. These data sources were linked by personal health number, covering all people registered for BC’s provincial health insurance who received a diagnosis of MDD during Fiscal Years 2008-09 to 2019-20. Data to calculate costs included medical service registration and demographic information [12], Medical Service Plan (MSP) payments to primary care physicians and specialists [13], records of hospitalizations from the Discharge Abstract Database (DAD) [14], emergency department (ED) visits from the National Ambulatory Care Reporting Systems (NACRS) [15], Vital Statistics deaths records [16], geography-based socioeconomic status quintiles [17] and records of Pharmacare payments for all prescriptions dispensed [18]. The median, before-tax income for quintiles in each census metropolitan area/census area was used to calculate the expenditure proportion of MDD-attributable spending to non-subsistence income, citing neighbourhood incomes from the PCCF + file published by Statistics Canada, version 7D [19].

#### Population, incidence, and prevalence

We conducted a retrospective cohort study using population data covering April 1, 2008, to March 31, 2020. The observational cohort included any person aged 19–99, with MDD that was newly diagnosed from April 1, 2015, to March 31, 2020. An incident case of MDD was defined by ≥1 hospitalization(s) with a most responsible diagnosis of MDD, or by ≥ two diagnoses in the physician claims within a one-year period, without having met the MDD criteria in any of the prior years back to April 1, 2008. We identified potential cases based on ICD-9 and 10 codes (see supplementary material), after excluding individuals who also met the diagnostic criteria for schizophrenia, schizoaffective or bipolar disorder with reference to a similar published method [20].

#### Derivations of costs

Total costs for health services used by patients who met the criteria for MDD were from the MSP physician payment data in the two-year follow-up period. The rate of ED visits was from combined MSP (where service location indicated ED) and NACRS data in the two-year follow-up period, and costs for those visits were estimated using an average daily ED visit cost of $405, accounting for standard ED physician fees ($96) [21] and facility costs ($309) combined per visit [22]. Costs attributed to ED use were calculated separately for visits that resulted in a hospital admission within 24-hour and standalone ED visits. The number of inpatient hospitalizations, total length of stay (LoS) and Resource Intensity Weighted costs (RIW; Case Mix Group and RIW times $6,618—the cost of a standard hospital stay in 2019–2020) were calculated [23]. Pharmaceutical expenses paid by PharmaCare and other payers were obtained directly from the PharmaNet data. The analysis included costs for all health services over the first two years of meeting the criteria for newly diagnosed MDD.

#### Cost outcomes

Health system costs were aggregated by treatment phase and refractory status. The acute phase of treatment was deemed to last 12 weeks, according to the commonly used definition in the clinical literature [24]. Weeks 13–52 and 53–104 were then separated into refractory and non-refractory status. Data for patients who met the criteria for refractory MDD were conservatively defined to include any patient with MDD prescribed more than five separate antidepressant drugs within the first two years after initially meeting the MDD criteria, to reflect sequential prescribing that did not result in remission [25]. Annual costs were discounted or inflated to costs in year 2020 using the average consumer price index (1.4%) for healthcare goods and services reported over years 2015-2019 [26].

### Costs not covered by the healthcare system

We searched the literature for studies reporting MDD-attributable costs paid by patients related to productivity costs (for either paid or unpaid work), informal caregiving, and out-of-pocket expenses, as defined by the SEG. No studies sufficiently captured all three cost categories. The terms: “societal + costs + depression” were selected to search PubMed for articles published between January 1, 2000, and April 1, 2021. We extracted the cost data from the literature for adults (age > 18) with MDD, excluding any articles that did not provide enough details to determine the severity of MDD, response to treatment or calculate the weekly cost. A data extraction tool was developed to justify inclusion/exclusion and evaluate the relevant considerations for calculating lost productivity and severity of MDD for which the cost data would be applied (see supplementary material). All costs were converted to 2020 Canadian dollars using purchasing power parities (PPP) relative to the US dollar with reference to historical indicators from the Organization of Economic Cooperation and Development, [27] then converted from US to Canadian dollars using the mean PPP indicators for services from Statistics Canada [28]. The cost data were separated into subgroups by MDD severity (no MDD, mild, moderate, severe) or treatment outcomes (MDD in remission or refractory to treatment). Finally, the total mean costs for each subgroup of MDD were calculated, net the mean costs to patients without MDD.

#### Expenditure proportion

The expenditure proportion was defined as the ratio of the cost patients pay to manage MDD (inclusive of: out-of-pocket costs, informal caregiving and costs attributed to lost productivity), relative to individualized non-subsistence income (monthly income, net the cost of living). We used the PCCF + file version 7D to determine the median, before-tax Quintile of Annual Income Per Person Equivalent (QABT-IPPE) income in each census metropolitan area or census area (CMA/CA) in BC. The QABT-IPPE comes from the household income reported to Statistics Canada, adjusted for household size. The cost of living was determined from WorkBC’s “cost of living calculator” with parameters to indicate home ownership, daily travel and household size imputed for each income quintile in each region based on the CMA/CA geography [29, 30]. Full details on the calculation and source data for the assumptions used are provided in the supplementary materials. The expenditure proportion was then calculated for each grade of MDD severity (no MDD, mild, moderate, or severe) or response to treatment (remission or refractory); by income quintile and region. When the expenditure proportion exceeded 40% of the QABT-IPPE, net the cost of living, this indicates financial hardship and if the proportion is below 40%, financial protection is achieved. For comparison with international indicators used by the WHO, we defined incidences of financial hardship across income groups and geographic areas by restricting the numerator of the expenditure proportion to out-of-pocket costs (OOPC). A two-way sensitivity analysis was used to test the median income assumption by imputing the lower (25th percentile) and higher (75th percentile) QABT-IPPE scenarios.

#### Distribution of financial protection

The distribution of financial protection across the province was measured with the slope index of inequality (SII) calculated as the coefficient from linear regression models for the expenditure proportion (outcome) between the lowest to highest income quintiles (ranking fractions) for each region and severity class for MDD. We used linear regression models in Stata IC version 16.1 to calculate SII, and considered a p-value below 0.1 significant to indicate a linear relationship between expenditure proportion and income quintiles. Values of SII equal to zero indicate a perfectly equitable distribution of financial protection between high- and low-income groups; higher or lower values of SII indicate inequality between the income groups. Positive values of SII indicate that financial protection is provided with advantages to lower income groups, while negative values of SII indicate advantages to high-income groups.

## Results

### Health system costs covered

**Fig. 1:**
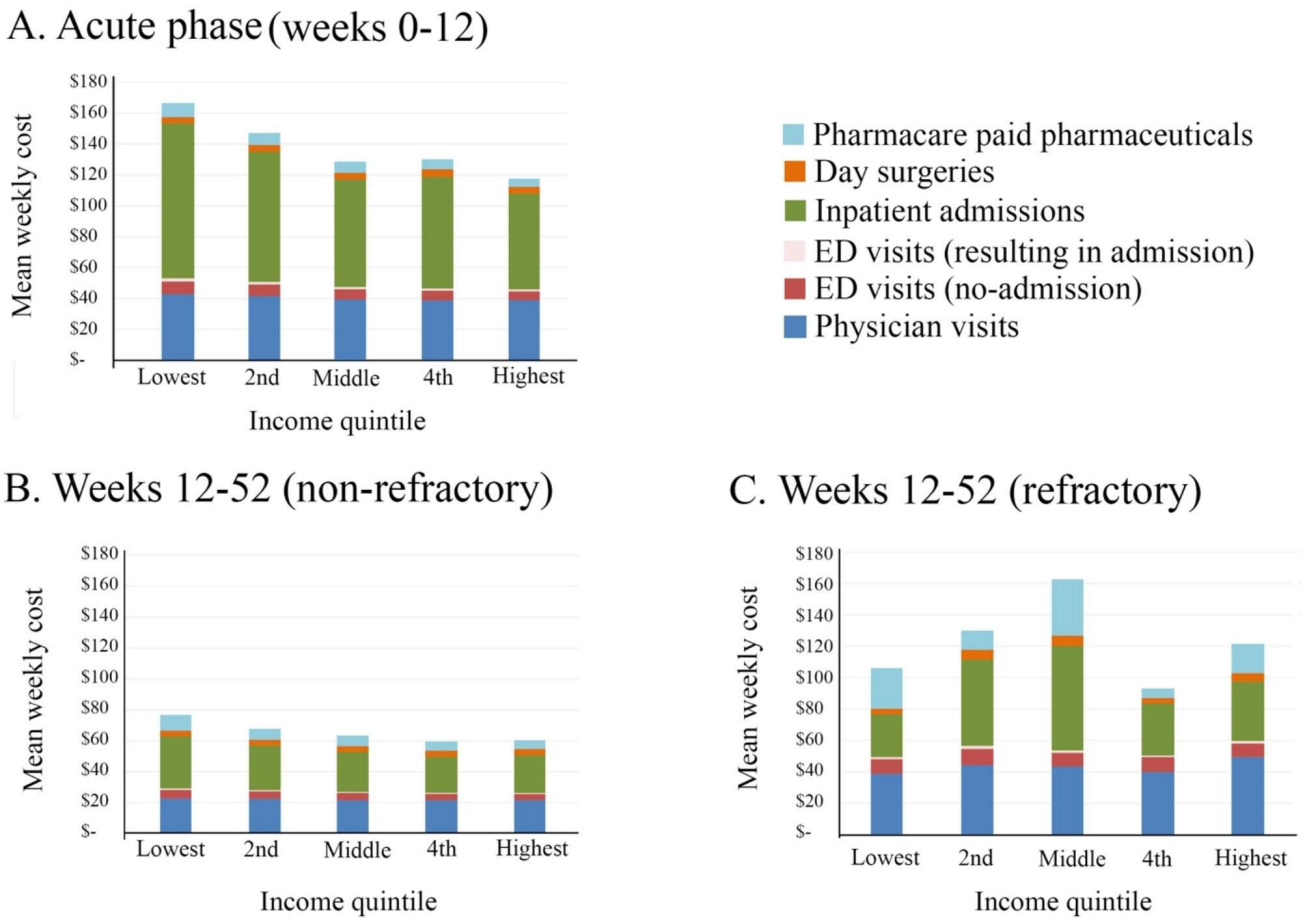
Healthcare system costs by income, treatment phase and refractory status **(A)** Weeks 1–12 for all patients receiving first-line treatment for acute phase MDD; weeks 13–52, patients with MDD that is **(B)** non-refractory to treatment or **(C)** refractory. Costs for prescriptions reflect the portion paid by the healthcare system. Physician costs include all family practice, medical, surgical, imaging and laboratory specialists

There were 250,855 patients in the study cohort who met the criteria for MDD, with slighter more females, an age distribution slightly above the provincial mean, the majority of patients lived in urban areas (86.6%) and slightly higher comorbidity scores in the cohort (Table 1). The average weekly health system costs totalled $138 for the first 12 weeks following a new diagnosis (Table 2). These costs were driven primarily by higher rates of inpatient admissions over the first 12 weeks compared with admission rates for the remaining 40 weeks in year one and weeks 53–104 for year two. Over the first 12 weeks the weekly incidence of admissions was 0.50% among the cohort. Over the remaining 40 weeks, the weekly rate of admissions among the cohort decreased to 0.26% and the total mean cost was $65/week, unless MDD was refractory to treatment, in which case the mean rate of admissions was 0.45% and total costs for patients with refractory MDD averaged $125/week. The weekly admission rate decreased to 0.21% for non-refractory MDD, (mean total weekly cost $55) and 0.33% for patients with refractory MDD in year two ($101/week). Among patients admitted for at least one hospitalization, the mean LoS was 1.36 days over weeks 0–12 however the mean LoS decreased to 0.28 days for patients labelled non-refractory and 0.42 for patients labelled refractory in weeks 13–52. When the total health system costs were disaggregated by income, costs were highest for patients in the lowest income group. High health system costs persisted among patients with MDD that was refractory to treatment (Fig. 1). Health system costs totaled $1.58 billion over the two years of follow-up for the cohort.

**Table 1:** MDD Cohort demographics

Characteristic	
N	250,855
Age Group, N (%)	
19–29	57,841 (23.1)
30–39	51,088 (20.4)
40–49	39,553 (15.8)
50–59	39,289 (15.7)
60–69	28,821 (11.5)
70–79	19,104 (7.6)
80–89	11,892 (4.7)
90–99	3,267 (1.3)
Age of diagnosis	
Mean (SD)	46.30 (18.78)
Median (IQR)	44 (30–60)
Sex/Gender, N (%)	
Female	148,952 (59.4)
Male	101,903 (40.6)
Urban/rural, N (%)	
Urban	217,296 (86.6)
Rural	30,158 (12.0)
Missing	3,401 (1.4)
Neighbourhood SES, N (%)	
1 Lowest	50,311 (20.1)
2	49,274 (19.6)
3	49,274 (19.6)
4	50,235 (20.0)
5 Highest	45,457 (18.1)
Missing	6,304 (2.5)
Elixhauser comorbidity score	
Mean (SD)	1.94 (1.41)
Median (IQR)	1 (1–2)
Diagnosis setting, N (%)	
Inpatient setting	4,997 (2.0)
Outpatient setting	245,858 (98.0)

**Table 2:** Health System Costs

N (%)	Term				
	First 12 weeks	Weeks 13–52		Weeks 53-104^1^	
	All	Non-refractory	Refractory	Non-refractory	Refractory
	250,855 (100%)	248,145 (98.9%)	894 (0.0%)	177,484 (70.8%)	677 (0.0%)
Average weekly cost (SD)	$137.84 (758)	$65.33 (263)	$125.23 (299)	$54.63 (184)	$101.19 (183)
Physician costs^2^ (SD)	$40.06 (80)	$21.74 (35)	$42.89 (47)	$18.84 (29)	$37.40 (36)
ED visits (no admission) (SD)	$6.88 (23)	$4.61 (13)	$9.26 (19)	$4.11 (11)	$8.17 (17)
ED visits (admission) (SD)	$1.63 (9)	$0.75 (4)	$1.53 (5)	$0.61 (3)	$1.16 (4)
Inpatient admissions (SD)	$77.76 (696)	$27.24 (226)	$45.82 (236)	$20.62 (146)	$28.23 (110)
Day surgeries (SD)	4.35 (36)	$3.83 (19)	$4.97 (23)	$3.63 (17)	$4.22 (14)
PharmaCare^3^(SD)	$7.16 (79)	$7.19 (61)	$20.76 (124)	$6.94 (53)	$22.02 (103)
**Total weekly cost (cohort)**	**$34,578,104**	**$16,210,556**	**$111,954**	**$9,696,227**	**$68,506**
**Total term cost (cohort)**	**$414,937,249**	**$648,422,240**	**$4,478,174**	**$504,203,824**	**$3,562,340**

^1^Year two costs were available for the portion of the cohort meeting the case definition prior to December 31, 2017

^2^FP+medical + surgical + imaging + laboratory specialist, patient is in any location except ED

^3^Amount PharmaCare paid for all drugs (including MDD drugs)

### Costs not covered by the health system

We retrieved 66 articles reporting costs to patients with MDD. Most of the studies (n = 45, 68%) were undertaken in European health systems, reporting on at least one of the cost categories of interest (n = 46, 69%); productivity costs were commonly reported among the included studies, 91% (n = 60), the majority (n = 39, 65%) reporting patient-level productivity indicators from trials, surveys or administrative data. Most studies reported on absenteeism and used the human capital method of valuation (n = 41, 68%). Eleven studies also used the friction cost method, which truncates productivity losses after a set period. The mean friction period used in these studies was 128 days. Other productivity changes, such as presenteeism, unpaid work and labour force participation, were rarely reported and thus excluded from our analysis. Most of the articles (n = 53, 80%) used cost-effectiveness methodology to support reimbursement decisions for MDD treatments. The weekly mean of the total patient-paid costs increased with MDD severity (no MDD, $69; mild, $269; moderate, $279; and severe MDD, $735) and refractory status (remission $107; refractory $1,021) (Table 3). Only 22 articles reported out-of-pocket expenses separately from the other patient-paid cost categories. The weekly mean for all grades of MDD combined was $43 and the weekly mean for patients without MDD or MDD in remission was $14.

**Table 3:** Costs not covered by the health system

Cost Category	Mean weekly cost by severity and treatment outcome ($, SD)					
	No MDD (below threshold)	Mild	Moderate	Severe	Remission	Refractory
Lost productivity	$17.38 (27)	$153.71 (130)	$168.85 (163)	$577.65 (429)	$ 80.15 (67)	$860.33 (667)
Informal caregiving	$35.39 (12)	$50.72 (38)	$58.78 (68)	$127.52 (161)	$10.98 (2)	$125.35 (150)
Out-of-pocket expenses	$16.68 (8)	$60.89 (82)	$47.46 (65)	$26.30 (21)	$12.21 (9)	$21.05 (2)
Antidepressant medication co-pays^1^	$0	$1.82 (4)	$1.82 (4)	$1.82 (4)	$1.82 (4)	$7.08 (11)
**Total**	**$69.45**	**$268.96**	**$278.73**	**$ 735.11**	**$ 106.98**	**$ 1,020.89**

^1^The amount patients pay for antidepressant drugs was available directly from administrative data using the patient-pays portion of the recorded drug costs, assuming that patients without MDD do not pay for antidepressants and that all grades of severity had similar medication costs for the first year

**Fig. 2:**
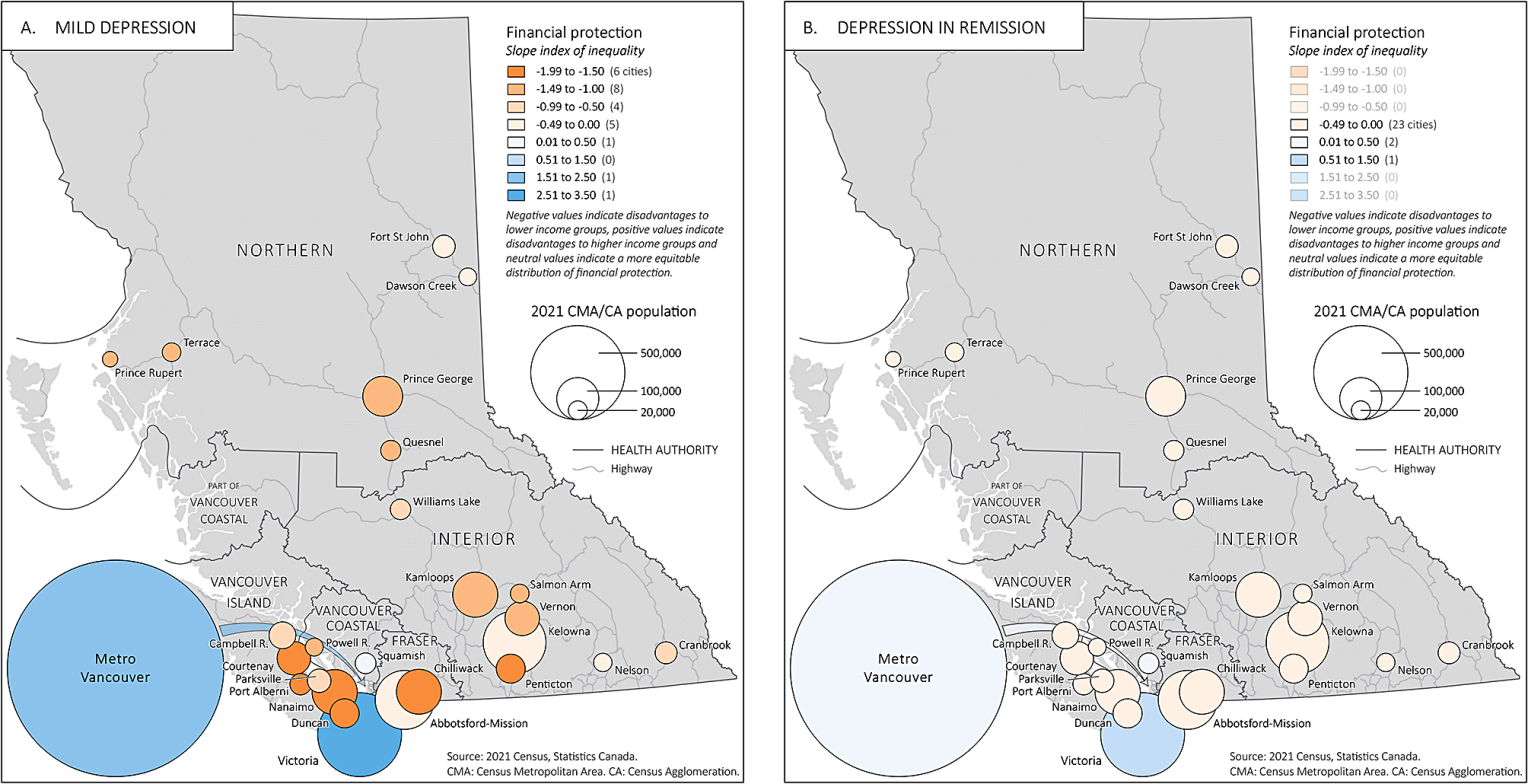
Slope index of inequality in financial protection. Slope index of inequality by region for **(A)** Mild MDD and **(B)** MDD in remission

### Distribution of financial protection

When the expenditure proportion was restricted to the oopc portion of patient-paid costs for comparison with the WHO threshold used to define financial hardship, the ratio of oopc to subsistence-adjusted QABT-IPPE exceeded the WHO-referenced 40% for homeowners in the two metropolitan cities (Vancouver (Q5 only) and Victoria (Q3-5)). The ratio was within the 30–40% range, for individuals in the lowest income groups in Nanaimo, Salmon Arm, Port Alberni, Chilliwack. The result was sensitive to median income; if the median QABT-IPPE was lower (25th percentile), seventeen of lowest income groups out of the twenty-six CMAs in study exceeded the WHO’s 40% threshold as well as homeowners in the higher income groups in five additional areas (Penticton, Abbotsford, Nanaimo, Kelowna, and Powell River). No incidences of financial hardship were observed in the higher income scenario (75th percentile).

Lost productivity accounted for the highest costs incurred by patients with MDD. The expenditure proportion of the total MDD-attributed costs paid by patients (total lost productivity, informal caregiving and oopc) exceeded 40% of non-subsistence QABT-IPPE in all income quintiles and regions in the province for any grade of MDD, but it fell below this threshold if MDD was in remission. The slope index of inequality measuring the distribution of financial protection across the income groups ranged from − 1.78 to 3.20 for mild MDD to -8.47 to 15.25 for MDD that is refractory to treatment. SII values were positive in areas close to major urban centres (Vancouver, Squamish, Abbotsford and Victoria), indicating inequality in financial protection from MDD for higher income groups due to house prices for homeowners in these areas, where monthly mortgage payments nearly exceeded the median monthly income. The relationship between the expenditure proportion and income rankings was linear in all CMA/CA regions except Vancouver, Squamish, Abbotsford, Kelowna and Nelson where high housing prices skewed the distribution of financial protection away from homeowners in higher income groups. Inequalities in financial protection resolved province-wide when MDD was in remission, (SII − 0.07 to 0.6) (Fig. 2).

## Conclusions

Our results show that the provincial health system paid >$1.5B over two years for patients with newly diagnosed MDD, comprising 2% of the province’s total $70B total health expenditures reported in 2018 and 2019 [22]. Over a quarter of the costs to manage MDD were incurred during the first 12 weeks of a new diagnosis. Costs not covered by the health system were estimated to total $3B to $12B over two years, depending on MDD severity and response to treatment. Low-income groups province-wide and middle-income homeowners in urban centres are at an increase risk of financial hardship. Income inequality in financial protection against the economic impact of MDD was observed for all grades of MDD in most regions of the province, but if MDD was in remission, patient costs decreased and the distribution of financial protection across income groups evened out. Access to early and effective treatment of MDD could have a significant impact both on the health budget and the distribution of financial protection offered by the healthcare system.

The current systems of coverage place extreme financial demands on patients managing even mild cases of MDD. Nearly a quarter of Canadians participating in the 2012 Canadian Community Health Survey who self-reported their MDD status, also self-reported that their treatment needs were unmet [5]. Other studies in Canada have also found that income, sex and formal educational attainment are disadvantages patients manage when accessing outpatient mental health services [31]. Collectively, our findings highlight the need to consider the societal perspective, when developing a mental health service coverage strategy. In 2006, a federal task force reporting progress on the objectives of Canada’s National Pharmaceutical Strategy recommended that a national threshold for catastrophic out-of-pocket spending be set to 5% of a patient’s income for coverage of prescription drug costs to prevent financial hardship due to the high cost of pharmaceuticals [32]. There is, however, no set threshold for non-pharmacological expenses related to treating mental illness; while these services are recommended, they are covered either through private insurance (where people have that) or paid for out-of-pocket in Canada.

Our analysis is limited by the need to rely on pooled data from patients managing MDD in other health systems. Because MDD is stigmatized and highly subject to discrimination, many patients are unlikely to feel safe about reporting their MDD status, resulting in gaps in our understanding of the extent of MDD-related expenditure that is not covered by the Canadian health system. Therefore, the costs and benefits for non-pharmaceutical interventions also remain largely invisible to analysts and these costs are often excluded from program evaluations. Embodiment of patient-paid costs would likely render our analysis an underestimate of the total costs of MDD that are not covered by Canada’s healthcare systems, however evidence from patient-reported self-paid costs attributed to MDD are unavailable at this time. Since MDD disproportionately affects women and younger adults, our findings underestimate financial hardship due to their increased risk of having lower income than the median QABT-IPPE. Data on regional income from PCCF + 7D source was also limited by representation in remote areas or areas with populations below 10,000, which may lead to inaccuracy in the expenditure proportion estimated for these areas.

We found that MDD-attributable costs are high from either the health system or patient’s perspective. Over a quarter of the health system costs of MDD occur during the first 12 weeks of a new diagnosis. Achieving remission early could mitigate financial hardship faced by patients, and even-out the distribution of financial protection across income groups. Rapid access to effective treatments thus presents a unique opportunity to save health systems costs and reduce inequities in cost sharing between the health system and patients managing MDD.

### Electronic supplementary material

Below is the link to the electronic supplementary material.

Supplementary Material 1

## Data Availability

Administrative data used in this study cannot be shared publicly because of privacy restrictions. Data are available for request through Population Data BC for researchers who meet the criteria for access to confidential data. Data used for the patient and family cost calculations were publicly available from Work British Columbia [29]. The extraction of these data and calculations are available from the corresponding author (Sonya Cressman) upon request.

## Declarations

### Disclaimer

Access to data provided by the Data Steward(s) is subject to approval but can be requested for research projects through the Data Steward(s) or their designated service providers. All inferences, opinions, and conclusions drawn in this publication are those of the author(s) and do not reflect the opinions or policies of the Data Steward(s).

## Abbreviations

MDD: major depressive disorder
SII: Slope index of inequality
WHO: World Health Organization
SEG: Stakeholder engagement group
ICD-9, ICD-10: International Classification of Disease version-9, -10
QABT-IPPE: Quintile of Annual Income Per Person Equivalent income
CMA: census metropolitan area
CA: census area
PCCF + 7D: Postal Code Conversion File version 7D
