# Supplementary material for: Costs of major depression covered / not covered in British Columbia, Canada
Source: BMC Health Serv Res. 2023 Dec 20;23:1446. doi: 10.1186/s12913-023-10474-y (PMC10734183; doi:10.1186/s12913-023-10474-y)
Supplement: Supplementary file 1 — Supplementary Material 1 [file 12913_2023_10474_MOESM1_ESM.docx]

Supplementary materials

S.1. Patient and stakeholder engagement

S.2. MDD cohort definition

Table S.1. ICD-9 and ICD-10-CA codes used to define the MDD cohort

S.3. Costs to patients and their families

S.3.1. Literature review

Table S.2 Data extraction tool

Figure S.3. Article attrition

S.3.2. MDD severity

Table S.3. Source data for costs to patients and their families

S.4. Subsistence income calculation

S.5. Expenditure proportion

Table S.4 Expenditure proportion by CMA/CA for mild MDD

Table S.5 Expenditure proportion by CMA/CA for MDD in remission

S.6. Slope index of inequality

Table S.6 SII values by CMA/CA

S.7. Supplemental References

S.1. Patient and stakeholder engagement

To gain an initial understanding of the type of societal costs attributable to MDD, we convened a meeting with the project’s Stakeholder Engagement Group (SEG) of 14 persons with diverse knowledge, skills, experience, and interest in improving outcomes for depression. The SEG’s input was used to design a data extraction tool, which was subsequently used to capture key data from published literature on MDD-attributable societal costs according to the severity of MDD and response to treatment. Several SEG members were also engaged as patient researchers in this study and the broader evaluation, contributing to study design, analysis of data, interpretation of results and dissemination of knowledge.

The SEG deliberated on the financial consequences of receiving treatment for MDD, response to treatment and one’s ability to afford the recommended treatments. They agreed that financial protection is a concern and noted that estimates of lost productivity due to MDD probably underestimate the true costs patients pay due to other workplace factors, such as stigma, and additional challenges in finding and securing an understanding employer and advancing professionally.

The SEG suggested that including informal (unpaid) costs in the analysis of financial risk protection is important since caregivers incur a significant burden of lost household income and time costs for supporting patients in gaining access to treatment or managing the illness without access to the recommended services. Patient researchers and SEG members offered insights that the additional out-of-pocket costs that are passed on to patients leave many of them without access. Non-reimbursed drugs and psychologist or counselling fees that rely on extended health benefits from employment and/or out-of-pocket payments are often underinsured or expire when patients are off from work for too long.

# Table S.1. ICD-9 and ICD-10-CA codes used to define the MDD cohort

| **ICD-9** | **ICD-10-CA** |
| --- | --- |
| 296 | F20.4 |
| 300 | F31.3-F31.5 |
| 309 | F32.x |
| 311 | F33.x |
| 50B | F34.1 |
|  | F41.2 |
|  | F43.2 |

x=1-9

|  |  |
| --- | --- |

S.3. Costs to patients and their families

S.3.1. Literature review

We search PubMed using the terms “societal” and “costs” and “depression”, between Jan 1, 2000- March 3, 2021 after trialling a number of search terms for costs paid by patients with MDD. A total of 425 articles were retrieved, and 154 were retained after screening by title. We included all empirical studies on adult patients with MDD that reported societal costs with MDD being at least one of the primary conditions; articles were excluded if they were not based on primary or secondary data (n=30), did not define MDD as at least one of the primary conditions (n=34), or if the details of the study did not include enough information to apply the patient cost calculation or assign a severity of MDD (n=24). A total of 66 studies met the criteria for inclusion, enabling application of the data extraction tool (Table S.1.) and patient and family cost method.

Table S.2. Data extraction tool

| Category | Field | Data | Description |
| --- | --- | --- | --- |
| Study Characteristics | population | String text | Define the patients, healthcare context, and assignment of grade of severity of MDD; used to apply inclusion and exclusion criteria |
|  | intervention | String text | Narrative synthesis to identify the cost data to reference. All cost data were extracted from the intervention arms |
|  | comparisons | String text | Comparison arms of interventional studies; required to distinguish between study arms so as to identify the intervention arm |
|  | outcomes | String text | Narrative synthesis of the context |
|  | study_design | String text | Narrative synthesis of the design and context |
|  | analysis_type | Categorical grouping of main instrument used | 1=CEA  2=Cost analysis  3=Productivity analysis  4=Other |
| Severity of depression | depression_subgroup | String text | Narrative description from the paper about the severity of depression included; used to assign a categorical grouping in depression_severity |
|  | depression_severity | Categorial grouping | Determined from severity_instrument  1=people without depression (no mdd)  2=no indication of severity  3=subthreshold  4=mild  5=moderate  6=severe  7=refractory |
|  | severity_instrument | Categorical grouping of main instrument used to label MDD-severity | Instrument used to assess severity:  1=Hamilton Depression Rating Scale (HAM-D)  2=Centre for Epidemiologic studies depression scale (CES-D)  3=Patient Heath Questionnaire-9 (PHQ-9)  4=Beck depression inventory-II (BD-II)  5=EuroQol-5 five dimension (EQ-5D)  6=Montgomery-Ashberg Depression Rating Scale (MADRS)  7=Hospital anxiety and depression scale (HADS)  8= Geriatric Depression Screening Scale (GDS-15)  9= Quick inventory of depressive symptomology (QIDS)  10=Severity of MDD described in the narrative text |
|  | no_mdd | Binary | Mean or median score of <10 points on HAM-D; 0 on CES-D; ≤ 4 on PHQ-9; ≤ 14 on BDI-II; ≥ 0.8 on EQ-5D; 0 to 6 on MADRS; ≤ 7on HADS; >5; -II; 0-4 on GDS-15; 0 – 5 on QIDS; or any narrative description indicating the complete absence of depressive symptoms for at least one year. |
|  | mild | Binary | Mean or median score between 10-13 on HAM-D; 1-3 on CES-D; 5-9 on PHQ9; on 14-19 on BDI-II; 0.57-0.79 on EQ-5D; 7 to 19 on MADRS; 8-10 points on HADS; 5-8 on GDS-15; 6-10 on QIDS; or narrative text describing simple MDD likely to respond to treatment. |
|  | moderate | Binary | Mean or median score between 14-17 on HAM-D; 4-6 on CES-D; 10-19 on PHQ9; on 20-28 on BDI-II; 0.52-0.56 on EQ-5D; 20 to 34 on MADRS; on 11–15 points on HADS; 9-11 on GDS-15; 11-15 on QIDS, or any narrative description indicating that MDD required secondary care by a psychiatrist, but no hospitalization was involved. |
|  | severe | Binary | Mean or median score >17 on HAM-D; ≥ 7 on CES-D; ≥ 20 PHQ9; ≥ 29 on BDI-II; <0.51 on EQ-5D; >34 on MADRS; ≥ 16 points on HADS ≥ 12 on GDS-15; on ≥ 16 QIDS or any narrative text indicating that MDD that resulted in >10 days of work per year, or required admission to hospital. |
|  | remission | Binary | Any MDD that has responded to one or more lines of treatment in the acute phase after having any severity of MDD within one year of MDD, regardless of severity. |
|  | refractory | Binary | Refractory included any MDD that did not respond to one or more lines of treatment in the acute phase, any “treatment resistant depression” |
|  | cost_analysis_group | Categorical | 1=cost-effectiveness analysis  2=productivity cost analysis  3=cost analysis only (no outcomes)  4=model for societal costs only  5=economic burden of disease  6=value of information analysis  7=other |
| Productivity | cost_category | Categorical, up to 3 entries | 1=absenteeism  2=presenteeism  3=informal caregiving  4=out-of-pocket expenses |
|  | lost_productivity_method | String | Narrative description of the method used to calculate lost-productivity |
|  | lost_productivity_valuation | Categorical | 1=Human capital approach  2=Friction cost approach  3= 1 and 2  4=WPA  5=other |
|  | friction_period | Continuous numerical | Friction period for calculating lost productivity (days) |
|  | labour_valuation | Categorical | Methodology for valuation of lost work time  1=GDP per capita  2=mean national/state/provincial incomes  3=patient income  4=insurance payments made 5=other methods  6= method not specified  7=minimum daily wage |
|  | labour_valuation_source | String | Narrative text describing the source of labour valuation data |
| Currency conversions | currency | String | International currency |
|  | currency_year | Numeric | Year of costs reported |
|  | ppp | Numeric | Purchasing power parity relative to USD in currency_year |
|  | fu_time_xx | Continuous | Follow-up time for studies reporting societal cost data in weeks, with xx= to either absenteeism, ic or oope |
|  | rate | Binary | 1=Absenteeism rate used to express costs as a function of wages  0=Mean costs of absenteeism directly reported |
| Costs to patients and their families | c_cost_category_fu_xx | Continuous | Mean costs for cost_category (1=absenteeism; 2=presenteeism; 3=informal caregiving 4=out of pocket costs at total follow_up_time in currency at currency_year; xx= grade of MDD (xx=no mdd, mild, moderate, severe, refractory, or remission) |
|  | c_cost_category_week_xx | Continuous | c_cost_category_fu_xx divided by fu_time; xx=grade of MDD |
|  | c_ cost_category _week_usd_xx | Continuous | c_cost_category_week_xx in international dollars (c_cost_category _week_xx/ppp); xx=grade of MDD |
|  | c_ cost_category _week_2020_cdn_xx | Continuous | c_cost_category_week_xx in 2020 Canadian dollars (c_cost_category _week_xx/ppp); xx=grade of MDD |

S.3.1. MDD severity groupings and response to treatment

The grade of severity of MDD was assigned to one or more patient groups from the extracted studies. We designated any data for “*people without depression”* if the study indicated that this group had not ever had depression or achieved a full remission after having completed acute phase treatment and at least one year of maintenance (*i.e.* 74 weeks since their last MDD episode). If the grade of severity of MDD was not disaggregated in either the reporting or implied by the context of the study, the study was excluded. We also excluded studies that included individuals who were at a high-risk of MDD or in a screening context. The severity of MDD was primarily assigned either by the mean or median score for a depression specific instrument; MDD was assumed to be moderate if treatment involved a psychiatrist without hospitalization and severe if there was a need for hospitalization or to take >10 days off work. Any article citing costs for MDD that did not respond to one or more lines of treatment in the acute phase, or any “treatment resistant depression” was grouped as refractory MDD, whereas if the MDD responded to treatment as indicated by >50% reduced HADS, HAMD, BDII or MADRS scores, the costs were classified as being in remission.

Table S.3. Source data for costs to patients and their families

| Article | Cost per week (2020 CDN$) | | | | | | | | | | | | | | | | | |
| --- | --- | --- | --- | --- | --- | --- | --- | --- | --- | --- | --- | --- | --- | --- | --- | --- | --- | --- |
|  | Lost productivity | | | | | | Informal caregiving | | | | | | Out-of-pocket expenses | | | | | |
|  | No mdd | mild | moderate | severe | remission | refractory | No mdd | mild | moderate | severe | remission | refractory | No mdd | mild | moderate | severe | remission | refractory |
| ^1^ |  | 113 | 380 | 451 | 74 |  |  |  |  |  |  |  |  |  |  |  |  |  |
| ^2^ |  |  |  | 754 |  |  |  |  |  |  |  |  |  |  |  | 9 |  |  |
| ^3^ |  |  |  | 1,146 |  |  |  |  |  |  |  |  |  |  |  |  |  |  |
| ^4^ |  |  |  |  |  |  |  |  |  |  |  |  |  |  |  |  |  |  |
| ^5^ |  |  |  |  |  | 2,053 |  |  |  |  |  |  |  |  |  |  |  |  |
| ^6^ |  |  |  |  |  |  | 42 | 62 | 87 |  |  |  |  |  |  |  |  |  |
| ^7^ |  |  |  |  |  | 373 |  |  |  |  |  |  |  |  |  |  |  |  |
| ^8^ |  |  |  |  | 70 |  |  |  |  |  |  |  |  |  |  |  |  |  |
| ^9^ |  |  |  |  |  |  | 21 | 57 |  |  |  |  | 20 | 24 |  |  |  |  |
| ^10^ |  |  | 30 |  |  |  |  |  |  |  |  |  |  |  |  |  |  |  |
| ^11^ |  | 408 | 413 | 751 |  |  |  | 23 | 9 | 28 |  |  |  |  |  |  |  |  |
| ^12^ |  |  |  |  |  | 479 |  |  |  |  |  | 231 |  |  |  |  |  |  |
| ^13^ | 52 |  |  |  | 145 | 292 |  |  |  |  |  |  | 23 |  |  |  | 24 | 24 |
| ^14^ |  |  |  |  | 77 | 185 |  |  |  |  |  |  |  |  |  |  |  |  |
| ^15^ |  |  |  | 772 | 236 | 805 |  |  |  |  | 12 | 20 |  |  |  | 20 | 12 | 20 |
| ^16^ |  | 335 |  |  |  |  |  |  |  |  |  |  |  |  |  |  |  |  |
| ^17^ |  | 79 |  |  |  |  |  | 112 |  |  |  |  |  | 223 |  |  |  |  |
| ^18^ |  |  |  |  |  | 2,051 |  |  |  |  |  |  |  |  |  |  |  |  |
| ^19^ | 0 |  | 138 |  | 30 |  |  |  |  |  |  |  |  |  |  |  |  |  |
| ^20^ |  |  |  |  | 4 |  |  |  |  |  |  |  |  |  |  |  |  |  |
| ^21^ |  | 34 | 21 | 80 |  |  |  |  |  |  |  |  |  |  |  |  |  |  |
| ^16^ |  |  | 128 |  |  |  |  |  |  |  |  |  |  |  | 5 |  |  |  |
| ^22^ |  |  |  |  | 54 |  |  |  |  |  | 12 |  |  |  |  |  | 6 |  |
| ^23^ | 0 | 108 |  |  | 55 |  |  | 35 |  |  |  |  |  |  |  |  |  |  |
| ^24^ |  | 28 |  |  |  |  |  |  |  |  |  |  |  | 9 |  |  |  |  |
| ^25^ |  |  |  | 61 |  |  |  |  |  | 42 |  |  |  |  |  | 57 |  |  |
| ^26^ |  |  | 49 |  |  |  |  |  |  |  |  |  |  |  |  |  |  |  |
| ^27^ |  | 135 |  |  |  |  |  |  |  |  |  |  |  |  | 133 |  |  |  |
| ^28^ |  | 357 | 399 |  |  |  |  |  |  |  |  |  |  |  |  |  |  |  |
| ^29^ | 0 | 49 | 120 | 415 |  |  |  |  |  |  |  |  |  |  |  |  |  |  |
| ^30^ |  |  | 91 |  |  |  |  |  |  |  |  |  |  |  |  |  |  |  |
| ^31^ |  |  | 599 |  |  |  |  |  | 10 |  |  |  |  |  | 128 |  |  |  |
| ^32^ |  |  | 17 |  |  |  |  |  | 102 |  |  |  |  |  | 1 |  |  |  |
| ^33^ |  |  |  | 117 |  |  |  |  |  |  |  |  |  |  |  |  |  |  |
| ^34^ |  |  | 449 |  |  |  |  |  |  |  |  |  |  |  |  |  |  |  |
| ^26^ |  |  | 58 |  |  |  |  |  |  |  |  |  |  |  |  |  |  |  |
| ^35^ |  |  | 40 |  |  |  |  |  |  |  |  |  |  |  |  |  |  |  |
| ^36^ |  |  | 23 |  |  |  |  |  | 10 |  |  |  |  |  | 10 |  |  |  |
| ^37^ |  |  | 49 |  |  |  |  |  |  |  |  |  |  |  |  |  |  |  |
| ^38^ |  |  |  |  |  |  |  |  |  | 313 |  |  |  |  |  |  |  |  |
| ^39^ |  |  |  | 720 |  |  |  |  |  |  |  |  |  |  |  |  |  |  |
| ^40^ |  |  |  |  | 195 |  |  |  |  |  |  |  |  |  |  |  |  |  |
| ^41^ |  |  | 143 |  |  |  |  |  | 184 |  |  |  |  |  |  |  |  |  |
| ^42^ | 0 | 167 |  |  | 76 |  |  |  |  |  |  |  |  |  |  |  |  |  |
| ^43^ |  | 18 | 40 |  | 15 |  |  |  |  |  |  |  |  |  |  |  |  |  |
| ^44^ |  | 153 | 318 | 1,177 |  |  |  |  |  |  |  |  |  |  |  |  |  |  |
| ^45^ |  | 278 |  |  |  |  |  | 3 |  |  |  |  |  |  |  |  |  |  |
| ^46^ |  |  | 104 |  | 57 |  |  |  |  |  |  |  |  |  |  |  |  |  |
| ^47^ |  |  |  | 509 |  | 856 |  |  |  |  |  |  |  |  |  |  |  |  |
| ^48^ |  |  |  |  |  |  | 43 | 94 |  |  |  |  | 7 | 13 |  |  |  |  |
| ^49^ | 52 |  |  | 295 | 146 | 1,406 |  |  |  |  |  |  |  |  |  | 20 |  | 20 |
| ^50^ |  |  |  |  |  |  |  |  | 9 |  |  |  |  |  |  |  |  |  |
| ^51^ |  |  |  |  | 11 |  |  |  |  |  | 9 |  |  |  |  |  | 6 |  |
| ^52^ |  |  | 199 |  |  |  |  |  |  |  |  |  |  |  |  |  |  |  |
| ^53^ |  |  | 62 |  |  |  |  |  |  |  |  |  |  |  | 8 |  |  |  |
| ^54^ |  |  | 47 |  |  |  |  |  |  |  |  |  |  |  |  |  |  |  |
| ^55^ |  |  | 214 |  |  |  |  |  |  |  |  |  |  |  |  |  |  |  |
| ^56^ |  |  | 48 |  |  |  |  |  |  |  |  |  |  |  |  |  |  |  |
| ^57^ |  |  | 202 |  |  | 341 |  |  |  |  |  |  |  |  |  |  |  |  |
| ^58^ |  |  | 80 |  |  | 189 |  |  |  |  |  |  |  |  |  |  |  |  |
| ^59^ |  |  |  |  |  | 584 |  |  |  |  |  |  |  |  |  |  |  |  |
| ^60^ |  |  |  | 45 |  |  |  |  |  |  |  |  |  |  |  |  |  |  |
| ^61^ |  |  |  | 1,374 | 37 | 1,678 |  |  |  |  |  |  |  |  |  |  |  |  |
| ^62^ |  | 43 |  |  |  |  |  | 20 |  |  |  |  |  | 73 |  |  |  |  |
| ^63^ |  |  | 436 |  |  | 751 |  |  |  |  |  |  |  |  |  |  |  |  |
| ^64^ |  |  |  |  |  |  |  |  |  |  |  |  |  | 24 |  |  |  |  |

S.4. Subsistence income

Subsistence income was calculated using the median, before tax income from QABT-IPPE PCCF-version 7D in each CMA/CA region for each income quintile. The median income was entered into the WorkBC calculator to determine consumption in terms of housing and transportation, living and personal, and basic taxes, extracted in February 2023; available at: <https://www.costofliving.workbc.ca>. Additional assumptions to derive cost-of-living were: two people with equivalent income contributing to shared expenses per household based on the average household size of 2.44 across Canada, that >62% of all British Columbian’s own a home in each region (Ownership Rates, Canada, Provinces, Territories and Metropolitan Areas, 1971–2011) and that most people in British Columbia live with at least one other person based on the range of average household size in BC across income quintiles (Q1=1.47, Q5=3.6) (https://www.cmhc-schl.gc.ca/en/professionals/housing-markets-data-and-research/housing-data/data-tables/household-characteristics/total-population-population-housing-need-by-sex-living-arrangement); we assumed home ownership if QABT-IPPE was above $50,000 for a condo, $60,000, $70,000 and $80,000 for a small, large and medium house. In Vancouver, a minimum income of $60,000 was required to own a condo, $70,000, $80,000 and $90,000 for houses, based on the mean annual before-tax income of homeowners in BC ($57,305) versus Vancouver ($62,505)^65^; A one-bedroom dwelling was assumed for the lowest income quintile in any region with > 15% of the housed population living in a one-bedroom dwelling, otherwise a 2 bedroom dwelling was assumed in accordance with data for provincial household dwellings^66^.

S.5. Expenditure proportion

Table S.4 Expenditure proportion by CMA/CA (region) for mild MDD

| Income  Quintile (lowest to highest) | Region | | | | | | | | | | | |
| --- | --- | --- | --- | --- | --- | --- | --- | --- | --- | --- | --- | --- |
|  | Cranbrook | Nelson | Penticton | Kelowna | Vernon | Salmon Arm | Kamloops | Chilliwack | Abbotsford | Vancouver | Squamish | Victoria |
| Q1 | 109% | 124% | 202% | 129% | 189% | 220% | 156% | 216% | 95% | 107% | 86% | 95% |
| Q2 | 109% | 146% | 197% | 159% | 152% | 50% | 117% | 150% | 187% | 123% | 142% | 86% |
| Q3 | 72% | 122% | 152% | 109% | 125% | 102% | 96% | 113% | 131% | 87% | 112% | 235% |
| Q4 | 68% | 121% | 95% | 115% | 109% | 93% | 74% | 88% | 115% | 59% | 116% | 347% |
| Q5 | 62% | 93% | 75% | 106% | 81% | 79% | 63% | 81% | 94% | 363% | 133% | 285% |

| Income  Quintile (lowest to highest) | Region | | | | | | | | | | | | | |
| --- | --- | --- | --- | --- | --- | --- | --- | --- | --- | --- | --- | --- | --- | --- |
|  | Duncan | Nanaimo | Parksville | Port Alberni | Courtenay | Campbell River | Powell River | Williams Lake | Quesnel | Prince Rupert | Terrace | Prince George | Dawson Creek | Fort St. John |
| Q1 | 195% | 213% | 120% | 223% | 197% | 148% | 152% | 131% | 195% | 170% | 188% | 170% | 93% | 75% |
| Q2 | 195% | 174% | 136% | 131% | 185% | 104% | 202% | 82% | 101% | 99% | 110% | 99% | 101% | 81% |
| Q3 | 110% | 102% | 114% | 90% | 126% | 101% | 135% | 80% | 80% | 90% | 97% | 90% | 87% | 66% |
| Q4 | 92% | 92% | 93% | 86% | 105% | 79% | 106% | 77% | 76% | 76% | 72% | 76% | 81% | 54% |
| Q5 | 70% | 80% | 82% | 74% | 83% | 68% | 79% | 62% | 64% | 72% | 64% | 72% | 59% | 50% |

Table S.5 Expenditure proportion by CMA/CA for MDD in remission

| Income  Quintile (lowest to highest) | Region | | | | | | | | | | | |
| --- | --- | --- | --- | --- | --- | --- | --- | --- | --- | --- | --- | --- |
|  | Cranbrook | Nelson | Penticton | Kelowna | Vernon | Salmon Arm | Kamloops | Chilliwack | Abbotsford | Vancouver | Squamish | Victoria |
| Q1 | 21% | 23% | 38% | 24% | 36% | 41% | 29% | 41% | 18% | 20% | 16% | 18% |
| Q2 | 20% | 27% | 37% | 30% | 29% | 9% | 22% | 28% | 35% | 23% | 27% | 16% |
| Q3 | 14% | 23% | 29% | 20% | 23% | 19% | 18% | 21% | 25% | 16% | 21% | 44% |
| Q4 | 13% | 23% | 18% | 22% | 20% | 17% | 14% | 16% | 22% | 11% | 22% | 65% |
| Q5 | 12% | 17% | 14% | 20% | 15% | 15% | 12% | 15% | 18% | 68% | 25% | 54% |

| Income  Quintile (lowest to highest) | Region | | | | | | | | | | | | | |
| --- | --- | --- | --- | --- | --- | --- | --- | --- | --- | --- | --- | --- | --- | --- |
|  | Duncan | Nanaimo | Parksville | Port Alberni | Courtenay | Campbell River | Powell River | Williams Lake | Quesnel | Prince Rupert | Terrace | Prince George | Dawson Creek | Fort St. John |
| Q1 | 37% | 40% | 23% | 42% | 37% | 28% | 29% | 25% | 37% | 32% | 35% | 32% | 18% | 14% |
| Q2 | 37% | 33% | 26% | 25% | 35% | 20% | 38% | 15% | 19% | 19% | 21% | 19% | 19% | 15% |
| Q3 | 21% | 19% | 21% | 17% | 24% | 19% | 25% | 15% | 15% | 17% | 18% | 17% | 16% | 12% |
| Q4 | 17% | 17% | 17% | 16% | 20% | 15% | 20% | 14% | 14% | 14% | 14% | 14% | 15% | 10% |
| Q5 | 13% | 15% | 15% | 14% | 16% | 13% | 15% | 12% | 12% | 14% | 12% | 14% | 11% | 9% |

S.6. Slope index of inequality

The slope index of inequality (SII) is a measure of deviation from a perfectly equitable distribution of financial protection. In this study, it is used to measure the inequity in MDD-attributed expenses paid by patients and their families, as a proportion of non-subsistence income. Non-zero values indicate inequality. Negative values of SII indicate relative disadvantages in the distribution of cost-sharing for the lowest income groups, while positive SII values indicate disadvantages in the highest income group. The later observation applies to four CMA regions at or near major urban centres where housing prices are high (Vancouver, Victoria, Squamish and Abbotsford) The proportion of BC’s population owning homes in these four regions represented 393,406 (20%) of the provinces housed population (2,041,835) in 2022.^66^ Regional differences in SII were mitigated province-wide if MDD was in remission (SII -0.07 to 0.60). Comprehensive details and open-source software for measuring health inequality with SII are publicly available from Equidade, a WHO-collaborating centre for health equity (<https://www.equidade.org/files>) or the Scottish Public Health Observatory (https://www.scotpho.org.uk/methods-and-data/measuring-health-inequalities/).

Table S.6. SII values by CMA/CA

| Region | Mild MDD | Moderate MDD | Severe MDD | Refractory MDD | MDD in remission |
| --- | --- | --- | --- | --- | --- |
| Cranbrook | -0.68 | -0.71 | -2.26 | -3.23 | -0.13 |
| Nelson* | -0.44 | -0.46 | -1.45 | -2.08 | -0.08 |
| Penticton | -1.78 | -1.86 | -5.93 | -8.47 | -0.33 |
| Kelowna* | -0.46 | -0.48 | -1.52 | -2.17 | -0.09 |
| Vernon | -1.30 | -1.36 | -4.33 | -6.19 | -0.24 |
| Salmon Arm | -1.20 | -1.26 | -4.00 | -5.71 | -0.23 |
| Kamloops | -1.14 | -1.20 | -3.80 | -5.43 | -0.21 |
| Chilliwack | -1.66 | -1.74 | -5.55 | -7.93 | -0.31 |
| Abbotsford* | -0.37 | -0.38 | -1.22 | -1.74 | -0.07 |
| Vancouver* | 2.24 | 2.35 | 7.49 | 10.70 | 0.42 |
| Squamish* | 0.35 | 0.36 | 1.16 | 1.65 | 0.07 |
| Victoria | 3.20 | 3.35 | 10.67 | 15.25 | 0.60 |
| Duncan | -1.76 | -1.84 | -5.86 | -8.38 | -0.33 |
| Nanaimo | -1.74 | -1.82 | -5.80 | -8.29 | -0.33 |
| Parksville | -0.60 | -0.62 | -1.99 | -2.84 | -0.11 |
| Port Alberni | -1.72 | -1.81 | -5.74 | -8.21 | -0.32 |
| Courtenay | -1.54 | -1.62 | -5.15 | -7.37 | -0.29 |
| Campbell River | -0.93 | -0.98 | -3.10 | -4.43 | -0.17 |
| Powell River | -1.22 | -1.28 | -4.06 | -5.81 | -0.23 |
| Williams Lake | -0.71 | -0.74 | -2.37 | -3.38 | -0.13 |
| Quesnel | -1.43 | -1.50 | -4.78 | -6.83 | -0.27 |
| Prince Rupert | -1.09 | -1.14 | -3.63 | -5.19 | -0.20 |
| Terrace | -1.43 | -1.50 | -4.79 | -6.84 | -0.27 |
| Prince George | -1.09 | -1.14 | -3.63 | -5.19 | -0.20 |
| Dawson Creek | -0.45 | -0.47 | -1.50 | -2.14 | -0.08 |
| Fort St. John | -0.38 | -0.40 | -1.27 | -1.82 | -0.07 |

* the relationship between the expenditure proportion and income is non-linear in these areas (p value >0.1). Caution should be exercised in interpreting these SII values.

S.7. Supplemental References

1. Wade AG, Toumi I, Hemels ME. A probabilistic cost-effectiveness analysis of escitalopram, generic citalopram and venlafaxine as a first-line treatment of major depressive disorder in the UK. *Curr Med Res Opin* 2005;21(4):631-42. doi: 10.1185/030079905x41462 [published Online First: 2005/05/19]

2. Vasiliadis HM, Lesage A, Latimer E, et al. Implementing Suicide Prevention Programs: Costs and Potential Life Years Saved in Canada. *J Ment Health Policy Econ* 2015;18(3):147-55.

3. Dewa CS, Hoch JS, Lin E, et al. Pattern of antidepressant use and duration of depression-related absence from work. *Br J Psychiatry* 2003;183:507-13. doi: 10.1192/bjp.183.6.507

4. Bang Madsen K, Vogdrup Petersen L, Plana-Ripoll O, et al. Early labor force exits in patients with treatment-resistant depression: an assessment of work years lost in a Danish nationwide register-based cohort study. *Ther Adv Psychopharmacol* 2020;10:2045125320973791. doi: 10.1177/2045125320973791 [published Online First: 2020/12/08]

5. Sluiter RL, Janzing JGE, van der Wilt GJ, et al. An economic model of the cost-utility of pre-emptive genetic testing to support pharmacotherapy in patients with major depression in primary care. *The Pharmacogenomics Journal* 2019;19(5):480-89. doi: 10.1038/s41397-019-0070-8

6. Langa KM, Valenstein MA, Fendrick AM, et al. Extent and cost of informal caregiving for older Americans with symptoms of depression. *Am J Psychiatry* 2004;161(5):857-63. doi: 10.1176/appi.ajp.161.5.857 [published Online First: 2004/05/04]

7. Ekman M, Granström O, Omérov S, et al. The societal cost of depression: evidence from 10,000 Swedish patients in psychiatric care. *J Affect Disord* 2013;150(3):790-7. doi: 10.1016/j.jad.2013.03.003 [published Online First: 2013/04/25]

8. Evans-Lacko S, Knapp M. Global patterns of workplace productivity for people with depression: absenteeism and presenteeism costs across eight diverse countries. *Soc Psychiatry Psychiatr Epidemiol* 2016;51(11):1525-37. doi: 10.1007/s00127-016-1278-4 [published Online First: 2016/09/27]

9. Luppa M, Heinrich S, Matschinger H, et al. Direct costs associated with depression in old age in Germany. *J Affect Disord* 2008;105(1-3):195-204. doi: 10.1016/j.jad.2007.05.008 [published Online First: 2007/06/15]

10. Müller G, Pfinder M, Schmahl C, et al. Cost-effectiveness of a mindfulness-based mental health promotion program: economic evaluation of a nonrandomized controlled trial with propensity score matching. *BMC Public Health* 2019;19(1):1309. doi: 10.1186/s12889-019-7585-4

11. Brettschneider C, Heddaeus D, Steinmann M, et al. Cost-effectiveness of guideline-based stepped and collaborative care versus treatment as usual for patients with depression – a cluster-randomized trial. *BMC Psychiatry* 2020;20(1):427. doi: 10.1186/s12888-020-02829-0

12. McCrone P, Rost F, Koeser L, et al. The economic cost of treatment-resistant depression in patients referred to a specialist service. *J Ment Health* 2018;27(6):567-73. doi: 10.1080/09638237.2017.1417562 [published Online First: 2017/12/26]

13. Ross EL, Soeteman DI. Cost-Effectiveness of Esketamine Nasal Spray for Patients With Treatment-Resistant Depression in the United States. *Psychiatr Serv* 2020;71(10):988-97. doi: 10.1176/appi.ps.201900625 [published Online First: 20200707]

14. Hornberger J, Li Q, Quinn B. Cost-effectiveness of combinatorial pharmacogenomic testing for treatment-resistant major depressive disorder patients. *Am J Manag Care* 2015;21(6):e357-65. [published Online First: 2015/08/08]

15. Fitzgibbon KP, Plett D, Chan BCF, et al. Cost-Utility Analysis of Electroconvulsive Therapy and Repetitive Transcranial Magnetic Stimulation for Treatment-Resistant Depression in Ontario. *Can J Psychiatry* 2020;65(3):164-73. doi: 10.1177/0706743719890167 [published Online First: 2019/12/06]

16. Holst A, Ginter A, Björkelund C, et al. Cost-effectiveness of a care manager collaborative care programme for patients with depression in primary care: economic evaluation of a pragmatic randomised controlled study. *BMJ Open* 2018;8(11):e024741. doi: 10.1136/bmjopen-2018-024741 [published Online First: 2018/11/14]

17. El Alili M, Schuurhuizen C, Braamse AMJ, et al. Economic evaluation of a combined screening and stepped-care treatment program targeting psychological distress in patients with metastatic colorectal cancer: A cluster randomized controlled trial. *Palliat Med* 2020;34(7):934-45. doi: 10.1177/0269216320913463 [published Online First: 20200429]

18. Sado M, Knapp M, Yamauchi K, et al. Cost-Effectiveness of Combination Therapy Versus Antidepressant Therapy for Management of Depression in Japan. *Australian & New Zealand Journal of Psychiatry* 2009;43(6):539-47. doi: 10.1080/00048670902873664

19. Hawthorne G, Cheok F, Goldney R, et al. The excess cost of depression in South Australia: a population-based study. *Aust N Z J Psychiatry* 2003;37(3):362-73. doi: 10.1046/j.1440-1614.2003.01189.x [published Online First: 2003/06/05]

20. Aragonès E, Sánchez-Iriso E, López-Cortacans G, et al. Cost-effectiveness of a collaborative care program for managing major depression and chronic musculoskeletal pain in primary care: Economic evaluation alongside a randomized controlled trial. *J Psychosom Res* 2020;135:110167. doi: 10.1016/j.jpsychores.2020.110167 [published Online First: 2020/06/20]

21. Krauth C, Stahmeyer JT, Petersen JJ, et al. Resource Utilisation and Costs of Depressive Patients in Germany: Results from the Primary Care Monitoring for Depressive Patients Trial. *Depression Research and Treatment* 2014;2014:730891. doi: 10.1155/2014/730891

22. Buntrock C, Berking M, Smit F, et al. Preventing Depression in Adults With Subthreshold Depression: Health-Economic Evaluation Alongside a Pragmatic Randomized Controlled Trial of a Web-Based Intervention. *J Med Internet Res* 2017;19(1):e5. doi: 10.2196/jmir.6587

23. Valenstein M, Vijan S, Zeber JE, et al. The cost-utility of screening for depression in primary care. *Ann Intern Med* 2001;134(5):345-60. doi: 10.7326/0003-4819-134-5-200103060-00007 [published Online First: 2001/03/10]

24. Kraepelien M, Mattsson S, Hedman-Lagerlöf E, et al. Cost-effectiveness of internet-based cognitive-behavioural therapy and physical exercise for depression. *BJPsych Open* 2018;4(4):265-73. doi: 10.1192/bjo.2018.38 [published Online First: 2018/07/31]

25. van Eeden M, Kootker JA, Evers SMAA, et al. An economic evaluation of an augmented cognitive behavioural intervention vs. computerized cognitive training for post-stroke depressive symptoms. *BMC Neurol* 2015;15:266-66. doi: 10.1186/s12883-015-0522-y

26. Goorden M, Vlasveld MC, Anema JR, et al. Cost-utility analysis of a collaborative care intervention for major depressive disorder in an occupational healthcare setting. *J Occup Rehabil* 2014;24(3):555-62. doi: 10.1007/s10926-013-9483-4 [published Online First: 2013/10/03]

27. Hemels ME, Kasper S, Walter E, et al. Cost-effectiveness analysis of escitalopram: a new SSRI in the first-line treatment of major depressive disorder in Austria. *Curr Med Res Opin* 2004;20(6):869-78. doi: 10.1185/030079904125003737 [published Online First: 2004/06/18]

28. Simons CJP, Drukker M, Evers S, et al. Economic evaluation of an experience sampling method intervention in depression compared with treatment as usual using data from a randomized controlled trial. *BMC Psychiatry* 2017;17(1):415. doi: 10.1186/s12888-017-1577-7 [published Online First: 2017/12/30]

29. Simon J, Harmer CJ, Kingslake J, et al. Value of monitoring negative emotional bias in primary care in England for personalised antidepressant treatment: a modelling study. *Evid Based Ment Health* 2019;22(4):145-52. doi: 10.1136/ebmental-2019-300109 [published Online First: 2019/09/29]

30. Bosmans JE, Brook OH, van Hout HP, et al. Cost effectiveness of a pharmacy-based coaching programme to improve adherence to antidepressants. *Pharmacoeconomics* 2007;25(1):25-37. doi: 10.2165/00019053-200725010-00004 [published Online First: 2006/12/29]

31. Meuldijk D, Carlier IV, van Vliet IM, et al. Economic Evaluation of Concise Cognitive Behavioural Therapy and/or Pharmacotherapy for Depressive and Anxiety Disorders. *J Ment Health Policy Econ* 2015;18(4):175-83. [published Online First: 2016/01/06]

32. Sitnikova K, Finch AP, Leone SS, et al. A brief cognitive behavioural intervention is cost-effective for primary care patients with medically unexplained physical symptoms compared to usual care. *J Psychosom Res* 2020;138:110217. doi: 10.1016/j.jpsychores.2020.110217 [published Online First: 2020/09/14]

33. Hemels ME, Kasper S, Walter E, et al. Cost-effectiveness of escitalopram versus citalopram in the treatment of severe depression. *Ann Pharmacother* 2004;38(6):954-60. doi: 10.1345/aph.1E010 [published Online First: 2004/04/29]

34. Kolovos S, Kenter RM, Bosmans JE, et al. Economic evaluation of Internet-based problem-solving guided self-help treatment in comparison with enhanced usual care for depressed outpatients waiting for face-to-face treatment: A randomized controlled trial. *J Affect Disord* 2016;200:284-92. doi: 10.1016/j.jad.2016.04.025 [published Online First: 2016/05/08]

35. Fernandez JL, Montgomery S, Francois C. Evaluation of the cost effectiveness of escitalopram versus venlafaxine XR in major depressive disorder. *Pharmacoeconomics* 2005;23(2):155-67. doi: 10.2165/00019053-200523020-00007 [published Online First: 2005/03/08]

36. Gerhards SA, de Graaf LE, Jacobs LE, et al. Economic evaluation of online computerised cognitive-behavioural therapy without support for depression in primary care: randomised trial. *Br J Psychiatry* 2010;196(4):310-8. doi: 10.1192/bjp.bp.109.065748 [published Online First: 2010/04/02]

37. Rubio-Valera M, Bosmans J, Fernández A, et al. Cost-effectiveness of a community pharmacist intervention in patients with depression: a randomized controlled trial (PRODEFAR Study). *PLoS One* 2013;8(8):e70588. doi: 10.1371/journal.pone.0070588 [published Online First: 2013/08/21]

38. Knapp M, Romeo R, Mogg A, et al. Cost-effectiveness of transcranial magnetic stimulation vs. electroconvulsive therapy for severe depression: a multi-centre randomised controlled trial. *J Affect Disord* 2008;109(3):273-85. doi: 10.1016/j.jad.2008.01.001 [published Online First: 2008/02/12]

39. van Baardewijk M, Vis PM, Einarson TR. Cost effectiveness of duloxetine compared with venlafaxine-XR in the treatment of major depressive disorder. *Curr Med Res Opin* 2005;21(8):1271-9. doi: 10.1185/030079905x56484

40. Pahlevan T, Ung C, Segal Z. Cost-Utility Analysis of Mindfulness-Based Cognitive Therapy Versus Antidepressant Pharmacotherapy for Prevention of Depressive Relapse in a Canadian Context: Analyse coût-utilité de la thérapie cognitive basée sur la pleine conscience contre la pharmacothérapie antidépressive pour prévenir la rechute de la dépression en contexte canadien. *Can J Psychiatry* 2020;65(8):568-76. doi: 10.1177/0706743720904613 [published Online First: 2020/02/08]

41. Bosmans JE, Schreuders B, van Marwijk HW, et al. Cost-effectiveness of problem-solving treatment in comparison with usual care for primary care patients with mental health problems: a randomized trial. *BMC Fam Pract* 2012;13:98. doi: 10.1186/1471-2296-13-98 [published Online First: 2012/10/12]

42. Maniadakis N, Kourlaba G, Mougiakos T, et al. Economic evaluation of agomelatine relative to other antidepressants for treatment of major depressive disorders in Greece. *BMC Health Serv Res* 2013;13:173. doi: 10.1186/1472-6963-13-173 [published Online First: 20130510]

43. Romero-Sanchiz P, Nogueira-Arjona R, García-Ruiz A, et al. Economic evaluation of a guided and unguided internet-based CBT intervention for major depression: Results from a multi-center, three-armed randomized controlled trial conducted in primary care. *PLoS One* 2017;12(2):e0172741. doi: 10.1371/journal.pone.0172741 [published Online First: 2017/02/28]

44. Annemans L, Brignone M, Druais S, et al. Cost-effectiveness analysis of pharmaceutical treatment options in the first-line management of major depressive disorder in Belgium. *Pharmacoeconomics* 2014;32(5):479-93. doi: 10.1007/s40273-014-0138-x [published Online First: 2014/02/21]

45. Geraedts AS, van Dongen JM, Kleiboer AM, et al. Economic Evaluation of a Web-Based Guided Self-Help Intervention for Employees With Depressive Symptoms: Results of a Randomized Controlled Trial. *J Occup Environ Med* 2015;57(6):666-75. doi: 10.1097/jom.0000000000000423 [published Online First: 2015/03/06]

46. Xie F, Despiegel N, Danchenko N, et al. Cost effectiveness analysis of escitalopram compared to venlafaxine and fluvoxamine in treatment of major depressive disorder. *Int J Psychiatry Clin Pract* 2009;13(1):59-69. doi: 10.1080/13651500802450506 [published Online First: 2009/01/01]

47. Nordström G, Despiegel N, Marteau F, et al. Cost effectiveness of escitalopram versus SNRIs in second-step treatment of major depressive disorder in Sweden. *J Med Econ* 2010;13(3):516-26. doi: 10.3111/13696998.2010.506371 [published Online First: 2010/08/12]

48. Bock JO, Brettschneider C, Weyerer S, et al. Excess health care costs of late-life depression - Results of the AgeMooDe study. *J Affect Disord* 2016;199:139-47. doi: 10.1016/j.jad.2016.04.008 [published Online First: 20160412]

49. Ross EL, Vijan S, Miller EM, et al. The Cost-Effectiveness of Cognitive Behavioral Therapy Versus Second-Generation Antidepressants for Initial Treatment of Major Depressive Disorder in the United States: A Decision Analytic Model. *Ann Intern Med* 2019;171(11):785-95. doi: 10.7326/m18-1480 [published Online First: 20191029]

50. Grochtdreis T, Brettschneider C, Bjerregaard F, et al. Cost-effectiveness analysis of collaborative treatment of late-life depression in primary care (GermanIMPACT). *Eur Psychiatry* 2019;57:10-18. doi: 10.1016/j.eurpsy.2018.12.007 [published Online First: 20190115]

51. Klein NS, Wijnen BFM, Lokkerbol J, et al. Cost-effectiveness, cost-utility and the budget impact of antidepressants versus preventive cognitive therapy with or without tapering of antidepressants. *BJPsych Open* 2019;5(1):e12. doi: 10.1192/bjo.2018.81 [published Online First: 2019/01/15]

52. Cocker F, Nicholson JM, Graves N, et al. Depression in working adults: comparing the costs and health outcomes of working when ill. *PLoS One* 2014;9(9):e105430. doi: 10.1371/journal.pone.0105430 [published Online First: 2014/09/03]

53. Chatterton ML, Mihalopoulos C, O'Neil A, et al. Economic evaluation of a dietary intervention for adults with major depression (the "SMILES" trial). *BMC Public Health* 2018;18(1):599. doi: 10.1186/s12889-018-5504-8 [published Online First: 2018/05/23]

54. Sado M, Yamauchi K, Kawakami N, et al. Cost of depression among adults in Japan in 2005. *Psychiatry Clin Neurosci* 2011;65(5):442-50. doi: 10.1111/j.1440-1819.2011.02237.x [published Online First: 2011/08/20]

55. Lavelle TA, Kommareddi M, Jaycox LH, et al. Cost-effectiveness of collaborative care for depression and PTSD in military personnel. *Am J Manag Care* 2018;24(2):91-98. [published Online First: 2018/02/21]

56. Seidl A, Danner M, Wagner CJ, et al. Estimation of Input Costs for a Markov Model in a German Health Economic Evaluation of Newer Antidepressants. *MDM Policy Pract* 2018;3(1):2381468317751923. doi: 10.1177/2381468317751923 [published Online First: 20180110]

57. Sobocki P, Ekman M, Agren H, et al. The mission is remission: health economic consequences of achieving full remission with antidepressant treatment for depression. *Int J Clin Pract* 2006;60(7):791-8. doi: 10.1111/j.1742-1241.2006.00997.x [published Online First: 2006/07/19]

58. Groessl EJ, Tally SR, Hillery N, et al. Cost-Effectiveness of a Pharmacogenetic Test to Guide Treatment for Major Depressive Disorder. *J Manag Care Spec Pharm* 2018;24(8):726-34. doi: 10.18553/jmcp.2018.24.8.726

59. OHTAC. <https://www.hqontario.ca/Evidence-to-Improve-Care/Health-Technology-Assessment/Reviews-And-Recommendations/Multi-gene-Pharmacogenomic-Testing-That-Includes-Decision-Support-Tools-to-Guide-Medication-Selection-for-Major-Depression>. 2021

60. Romeo R, Patel A, Knapp M, et al. The cost-effectiveness of mirtazapine versus paroxetine in treating people with depression in primary care. *Int Clin Psychopharmacol* 2004;19(3):125-34. doi: 10.1097/00004850-200405000-00002 [published Online First: 2004/04/27]

61. Soini E, Hallinen T, Brignone M, et al. Cost-utility analysis of vortioxetine versus agomelatine, bupropion SR, sertraline and venlafaxine XR after treatment switch in major depressive disorder in Finland. *Expert Rev Pharmacoecon Outcomes Res* 2017;17(3):293-302. doi: 10.1080/14737167.2017.1240617 [published Online First: 2016/10/23]

62. Bosmans JE, van Schaik DJ, Heymans MW, et al. Cost-effectiveness of interpersonal psychotherapy for elderly primary care patients with major depression. *Int J Technol Assess Health Care* 2007;23(4):480-7. doi: 10.1017/s0266462307070572 [published Online First: 2007/10/17]

63. von Knorring L, Akerblad AC, Bengtsson F, et al. Cost of depression: effect of adherence and treatment response. *Eur Psychiatry* 2006;21(6):349-54. doi: 10.1016/j.eurpsy.2006.04.005 [published Online First: 20060613]

64. Wiley-Exley E, Domino ME, Maxwell J, et al. Cost-effectiveness of integrated care for elderly depressed patients in the PRISM-E study. *J Ment Health Policy Econ* 2009;12(4):205-13.

65. Canada S. Real average total household incomes before taxes for homeowner households in Canada, the provinces and selected Census Metropolitan Areas (CMAs). This table gives housing professionals a summary of changes in before-tax household income for homeowners from 2006 to 2017. . *Survey of Labour and Income Dynamics 2006 – 2011* Canadian Income Survey 2012 – 2017

66. Canada S. Structural type of dwelling by tenure: Canada, provinces and territories, census metropolitan areas and census agglomerations Table 98-10-0239-01 [cited 2023 April 27]. Available from: <https://www150.statcan.gc.ca/t1/tbl1/en/tv.action?pid=9810023901>.
